# The Roles of Dopamine and Hypocretin in Reward: A Electroencephalographic Study

**DOI:** 10.1371/journal.pone.0142432

**Published:** 2015-11-23

**Authors:** Armand Mensen, Rositsa Poryazova, Gordana Huegli, Christian R. Baumann, Sophie Schwartz, Ramin Khatami

**Affiliations:** 1 Department of Sleep Medicine, Clinic Barmelweid, 5017 Aargau, Switzerland; 2 Department of Neurology, University Hospital Zurich, 8006 Zurich, Switzerland; 3 Department of Neuroscience, University of Geneva, 1211 Geneva, Switzerland; Hospital General Dr. Manuel Gea González, MEXICO

## Abstract

The proper functioning of the mesolimbic reward system is largely dependent on the neurotransmitter dopamine. Recent evidence suggests that the hypocretin system has significant projections to this reward system. We examined the distinct effects of reduced dopamine or reduced hypocretin levels on reward activity in patients with Parkinson’s disease, dopamine deficient, as well as patients with narcolepsy-cataplexy, hypocretin depleted, and healthy controls. Participants performed a simple game-like task while high-density electroencephalography was recorded. Topography and timing of event-related potentials for both reward cue, and reward feedback was examined across the entire dataset. While response to reward cue was similar in all groups, two distinct time points were found to distinguish patients and controls for reward feedback. Around 160ms both patient groups had reduced ERP amplitude compared to controls. Later at 250ms, both patient groups also showed a clear event-related potential (ERP), which was absent in controls. The initial differences show that both patient groups show a similar, blunted response to reward delivery. The second potential corresponds to the classic feedback-related negativity (FRN) potential which relies on dopamine activity and reflects reward prediction-error signaling. In particular the mismatch between predicted reward and reward subsequently received was significantly higher in PD compared to NC, independent of reward magnitude and valence. The intermediate FRN response in NC highlights the contribution of hypocretin in reward processing, yet also shows that this is not as detrimental to the reward system as in Parkinson’s. Furthermore, the inability to generate accurate predictions in NC may explain why hypocretin deficiency mediates cataplexy triggered by both positive and negative emotions.

## Introduction

Dopamine is the primary neuro-modulatory force in the mesolimbic reward system [1]. Although Parkinson’s Disease (PD) is primarily characterized by a variety of motor-related disturbances, reward-related impairments are also commonly observed at both the clinical and neurophysiological levels [2–4]. Recent findings suggest important influences from several other neurotransmitters including hypothalamic hypocretin (HCRT or orexin) [5]. HCRT has been mainly associated with the arousal system [6,7]. However, HCRT neurons are ideally situated in the posterior hypothalamus to interact with dopamine neurons found in the ventral tegmental area (VTA) [8]. Animal research has shown that administration of HCRT into the VTA results in a direct increase in levels of dopamine which subsequently activates the entire mesolimbic system [9,10].

A lack of HCRT is strongly associated with narcolepsy-cataplexy (NC). The disease is clinically characterized by excessive daytime sleepiness, fragmented night-time sleep, and sudden, usually brief, periods of muscle atonia (cataplexy) triggered by strong emotional experiences [11]. We previously demonstrated reduced activity of the VTA while NC patients played a delay incentive task during the anticipation of reward as well as reduced activity of the nucleus accumbens combined with greater activity in dorsal striatum and amygdala during reward delivery, compared to healthy controls (HC), suggesting altered reward processing in NC [12]. However, it remains unclear whether HCRT and dopamine deficiency, in NC and PD patients respectively, may affect distinct facets of reward processing.

To better understand the respective contributions of HCRT and dopamine to reward processing, we investigated neural responses to reward feedback in patients with PD, NC, and HC, using electroencephalography (EEG). A tool we have previously demonstrated to be effective in these populations, and sensitive to potential differences in reward-related tasks [13]. We adapted a delay incentive reward task that we used in our previous on NC patients using functional magnetic resonance imaging (fMRI) [12]. Our aim was to characterize, for the first time in the same study, the direct neurophysiological responses and timing of brain responses to rewards in NC, PD patients, and HC. High-density EEG, with its high a temporal resolution can provide unique information about the timing of neural processes, with sufficient spatial information to differentiate topographical maps and thus underlying neural sources. In particular, previous research using EEG has shown several components related to reward processing, chief among them the feedback related negativity (FRN) component and the P300 component [14].

## Methods and Materials

### Participants

Table 1 provides demographic information HC, NC and PD groups. EEG was recorded from 12 participants for each group in the University Hospital Zurich and Clinic Barmelweid. HCRT in the cerebrospinal fluid could be obtained in 6 of the 12 patients and all showed undetectable levels. HLA typing was positive for HLA-DQB1*0602 in all 11 NC patients tested (one patient could not be tested). Parkinson’s disease was diagnosed using international criteria [15]. Mean disease duration, time since prominent symptoms (not diagnosis), for the NC group was 18.9 years (se = 4.3), while for the PD group it was 7.0 (se = 1.5). Each participant signed an informed consent form prior to the start of the experiment. The study was independently approved by both the cantonal ethical commissions of Zurich and Aarau. Patients were asked to participate in the study during their routine check-ups at the clinic and generally performed the experiment within a month of the visit. No explicit inclusion criteria was used for the patient group aside from their clear medical diagnosis according to international criteria and their willingness to participate.

**Table 1:** Participant demographic overview including statistical analysis of population differences.

	Healthy Controls	Narcolepsy Cataplexy	Parkinson’s Disease	Statistics
**Age**	34.1	40.2	68.7	F_2,33_ = 31.87
	(*4*.*1*)	(*3*.*0*)	(*2*.*3*)	p < 0.001
**Gender**	6 Male	5 Male	7 Male	χ^2^_36_ = 0.67
	6 Female	7 Female	5 Female	p = 0.717
**ESS**	5.0	16.9	7.3	F_2,33_ = 30.23
	(*1*.*0*)	(*1*.*4*)	(*1*.*2*)	p < 0.001
**BDI**	2.2	10.6	7.9	F_2,33_ = 6.99
	(*0*.*7*)	(*2*.5)	(*1*.*0*)	p = 0.003
**Medication**	None (12)	None (4), Sodium Oxybate (5; of 1.5–4.5g))*, Modafinil (4; 100mg)	None (1), Levodopa (11; of 62.5-250mg), Clonazepam (3; of 0.25–0.5mg)^+^	

Participant demographics and statistical differences. ESS = Epworth Sleepiness Score, BDI = Beck’s Depression Inventory, ULN = Ullanlinna Narcolepsy Scale.

*1 NC patient took both Sodium Oxybate and Modafinil.

^+^3 Parkinson’s patients took a combination of Levodopa and Clonazepam.

As shown in Table 1 and expected from the distinct pathologies, NC and PD patient groups differed in several measures. Given our previous study in fMRI, HC were selected and matched for age and gender with respect to the NC group alone. Crucially, due to both ethical and clinical restrictions, 8 of the 12 NC patients maintained their regular level of medication during the experiment. All dosages of modafinil were of 100mg while sodium oxybate ranged from 1.5g, 2.25g, or 4.5g. Furthermore, 11 of the 12 PD patients kept to their normal dosages of medication, all with dopaminergic properties, and one patient being drug-naive. Dosages of levodopa ranged from 62.5mg to 250mg, and clonazepam dosages were either 0.25mg or 0.5mg. Although the particular effects of continuing dopaminergic treatment in PD patients are fairly complex and difficult to maintaining patients medication reduces the likelihood of complete apathy in this patient group, and provides a more realistic everyday perspective as the vast majority of PD patients do indeed receive treatment.

### Task

**Fig 1:**
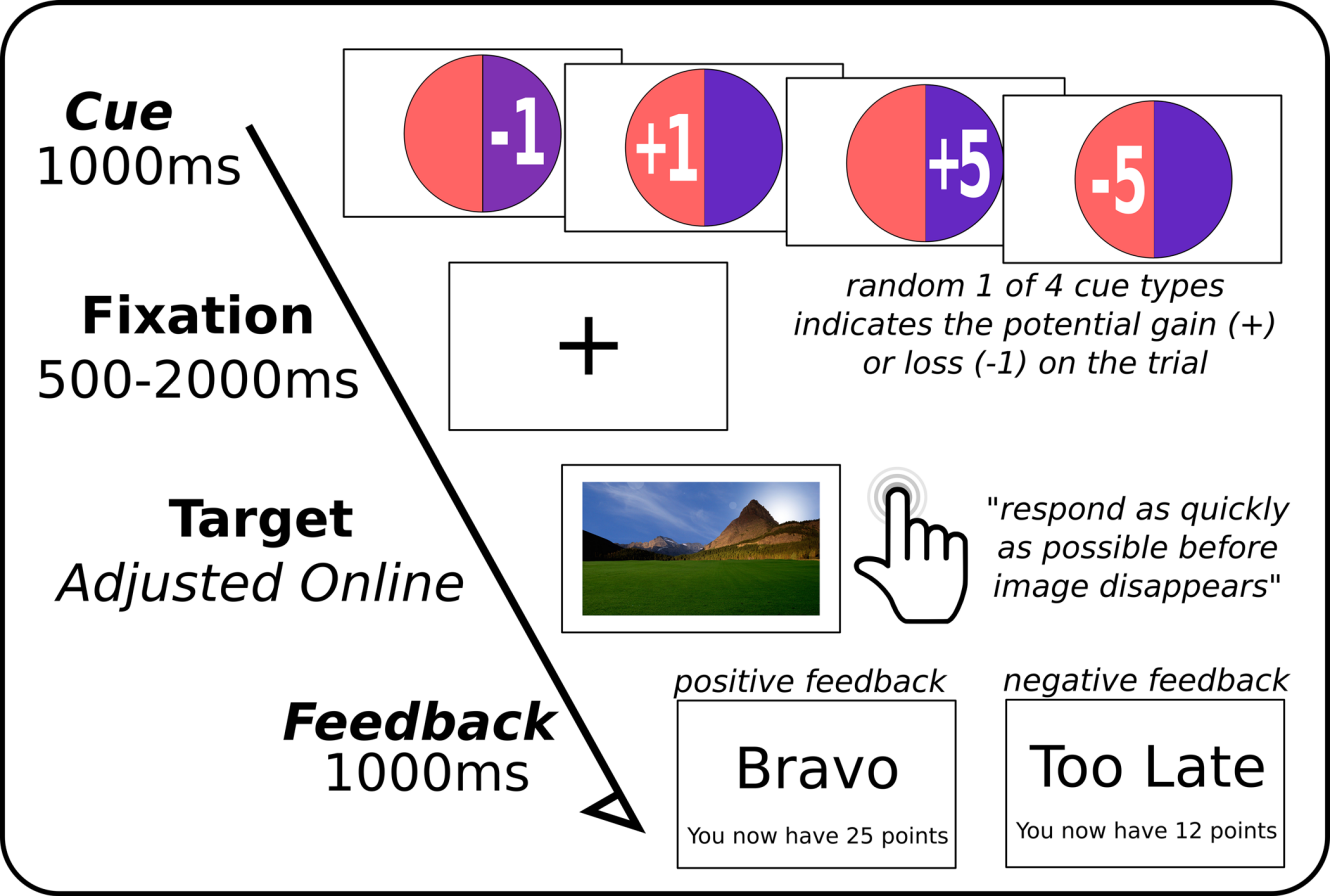
Overview of experimental task. One of the four possible cues appeared on the screen followed by a fixation cross for a random interval. Colour scheme was random and irrelevant for the current task. Participants were asked to press the button as quickly as possible once the landscape picture (one of a random set of ten), was presented on the screen. This was followed by a feedback display as to whether or not they were successful in the task and their current point total.

Fig 1 provides an overview of the experimental conditions. The task was a modified version of Knutson et al [16]. The goal of the reaction time task was to accumulate the maximum amount of points by the end of the game. Each trial presents the opportunity to either win points or not lose points on a correct response. A single trial can be split into 4 parts: a cue indicating the potential gains or losses of that trial; a fixation screen; a target screen where participants had to respond as quickly as possible; and finally a feedback screen indicating to the participant whether or not they were successful. At the onset of each trial, participants were presented with a circle for approximately 1 second with either a ‘5’ or ‘1’ representing high and low cues, with a preceding ‘+‘ or ‘-’ sign for potential gains and losses, respectively. This first screen thus cued participants to the magnitude of potential gains and losses to be expected from their success or failure in the task. Following a short interval (randomly jittered between 0.5 and 2 seconds), where participants fixated on a cross in the middle of the screen, a large picture of a landscape appeared (randomly selected from ten available). To successfully complete the trial, participants were required to push a button as quickly as possible while the landscape was present on the screen. The consequence of a successful reaction time on positive trial trial would be to add the appropriate amount to their total, while for negative trials, success would mean their current point total would not change. In this way, participants only lost points on a failures for -1/5 trials, and gained nothing on failures of +1/5 trials.

The duration of the presentation of the landscape was adjusted online; correct responses would shorten by 33 ms, while incorrect (late) responses would lengthen presentation time of the subsequent target by 33 ms. This online adjustment was run independent of trial type. This ensured a convergence around the participant-specific mean reaction time, and also an approximate 50% chance for a successful trial and subsequent positive feedback. Feedback consisted of a short confirmation phrase “Bravo”, or “Too late”; followed by the current total of points.

Participants performed 200 trials in 4 blocks of 50 trials, with a short break (about one minute at the participant’s discretion), between blocks so that participants could rest. The experimental task took approximately 12 minutes to complete. To ensure exact timing of stimulus presentation and markers on the EEG recording, the main task was programmed using Psychtoolbox [17], a Matlab toolbox (The MathWorks, Inc., Natick, Massachusetts, United States). This task is available for public download on the Github repository along with the data. Participants were seated comfortably with their head supported by a chin-rest (to minimize movement and fatigue), at a viewing distance of 60 cm from the screen. The screen had a resolution of 1920x1280, a diagonal size of 61 cm, a refresh rate of 60 Hz, a screen response time of 2ms, and a mean luminance level of 300cd/m2.

### EEG Recording and Preprocessing

EEG was recorded from 125 sites on the scalp using a HydroCel Geodesic Sensor Net by Electrical Geodesics, Inc; EGI [18]), and recorded at 1000Hz. Impedances for all electrodes were kept below 50kΩ. All EEG pre-processing was performed using Brain Vision’s Analyzer (version 2; Brain Products, Munich, Germany). Dataset analysis followed a set pipeline with appropriate manual participant-specific adjustments. Band-pass filters were applied to the continuous signal between 1 and 30 Hz using a modest 12/24 dB slope, and a notch filter at 50 Hz was used to remove main-power noise. Data were then down-sampled to 250 Hz, bad channels were manually removed, and classic independent component analysis (ICA) over the entire dataset was performed to manually remove components of the EEG associated with eye blinks and movements; electrocardiography signals (especially present in posterior electrodes); electromyography artifacts (predominantly over temporal electrodes); and any rhythmic tremor related artifacts in the PD group (examples presented in *supporting information*). Bad channels were then topographically interpolated using 3D splines (no more than 5/125 per participant [19]; all channels were then re-referenced to the average activity over all electrodes; and the data were then semi-automatically checked for any remaining artifacts under the following criteria: maximal allowed voltage step of 25 μV/ms; maximal absolute difference of 75 μV/ms over 200 ms window; EEG within -150 μV and 150 μV in amplitude. Finally, individual ERPs were created from 200 ms prior to the cue or feedback presentation up to 800 ms post-event; resulting in 1-second long ERPs for all 125 channels and 250 sample-points.

### Behavioral Assessment

Behavioral assessment was performed on the participants’ base reaction times and percentages of correct trials. The Epworth Sleepiness Scale measuring subjective long term sleepiness (ESS) and age are inherently linked to the group differences (i.e. NC patients have a higher ESS and PD patients were older), and thus correlate highly with group differences (Group and Age: F_2_,_33_ = 31.925, p < 0.001, R^2^ = 0.659; Group and ESS: F_2_,_33_ = 30.226, p < 0.001, R^2^ = 0.647). As such, we cannot include either measure directly into an analysis of covariance (ANCOVA), as this clearly violates the assumption of independence of predictors [20,21]. However, we can compute new constructs for ESS and Age, ESS* and Age* respectively, by specifically removing the covariance they have with the group. This is done by simply subtracting the sub-group mean from individual scores. In this way, we are left with covariates that are by definition orthogonal to the group independent measure, but nonetheless reflect within-group variation in their scores.

### ERP Analysis

Statistical analyses of the ERPs were conducted on all 125 channel for the entire 1s duration of the ERP dataset automatically using a threshold-free cluster-enhancement (TFCE) followed by maximum permutation statistics controlling for the multiple comparisons of all channels-sample pairs [22]. Briefly, a three-by-two analysis of variance corresponding to the group and condition factors was performed for all channels and time samples in a mass-univariate approach. The resulting F-values were then subjected to the TFCE adjustment which involves searching each channel-sample pair neighborhood for above threshold F-values over several consecutive thresholds from zero to the maximum F-value found (thus making it essentially threshold free). In this way, statistical differences that are well supported by their neighbors may be enhanced, and those at which neighboring points differ may be suppressed. This process was repeated 5000 times using randomly permuted datasets of the original subjects and conditions to create an empirical null-distribution from which an individual and unique p-value can be calculated for each channel-sample pair using an openly available toolbox for Matlab [23]. This technique has been shown to be statistically valid, requiring essentially no assumptions to be made about the structure or distribution of the data; and optimally sensitive to the various signal types found in EEG (e.g. intense but focal differences as well as distributed differences across the scalp). For this approach, referred to as TFCE_ANOVA_, permutations were done on the original data in a two-step process: first the within-subject levels were randomized (e.g. high vs low magnitude cues); then the entire participant dataset of all conditions was randomized between the groups [24].

In accordance with our experimental aims, we tested the ERPs from both the cue and feedback conditions. We primarily examined and report on the distinction between real gain (a positive outcome following a positive cue), and real loss (a negative outcome following negative cue), feedback; however all condition types were examined and any significant difference to the primary contrast are reported. In this way, both reward anticipation and reward processing can be examined in the ERP without any a-priori expectations regarding scalp location or time points of interest. Planned contrasts were conducted by means of more classic analysis of covariance (ANCOVA) taking significant regions of interest in both channel space and time found in the complete TFCE_ANOVA_ procedure, while also using participants’ ages and sleepiness scores as additional covariates in the model.

## Results

### Behavioral

An ANCOVA was conducted on participants’ reaction times with *Run (1 to 4)*, *Valence (*positive (+5 or +1) and negative (-5 or -1)) and *Magnitude (*high (+5 or -5) and low (+1 or -1)) as within-subject variables; *Group* as between subjects variable; and group-orthogonal constructs of the participants ESS* and Age* as covariates.

Although HCs had slightly faster mean reaction times than either of the patient groups (mean_HC_ = 281 ms, se = 18 ms; mean_NC_ = 304, se = 16 ms; mean_PD_ = 306 ms, se = 16 ms), the ANCOVA did not reveal any effect of *Group* (F_2,28_ = 1.093, p = 0.349), or *Valence* of the preceding cue (F_1,28_ = 0.651, p = 0.426), nor of cue *Magnitude* (F_2,33_ = 0.632, p = 0.433). There was however a main effect of *Run* (F_3,84_ = 8.857, p < 0.001); in that all participants were significantly slower on the first block of trials (mean = 328 ms, se = 9 ms), than the three subsequent blocks (mean = 288 ms, se = 13m). Within-group variability in age (Age*) explained a significant portion of participants’ reaction times (F_1,28_ = 4.836, p = 0.036); in that the older the participant, the slower their reaction time.

For the number of correct responses, we found a significant main effect of *Valence* (F_1,28_ = 6.094, p = 0.020), in that participants were generally more successful for positively cued trials (mean_negative_ = 22.63, se = 0.768; mean_positive_ = 25.08, se = 0.867). However, this difference was not specifically affected by any group difference (F_2,28_ = 0.733, p = 0.490), nor by the *Magnitude* of the cue (F_1,28_ = 2.729, p = 0.110). The three factors together also did not produce a significant interaction on the number of correct responses (F_2,33_ = 0.1234, p = 0.305). No other factors or interactions reached trend or significance levels. As expected from the online adjustment of target presentation times, groups also did not differ significantly in their point totals throughout the experiment (F_2,33_ = 0.685, p = 0.511).

### EEG: Cue Event

**Fig 2:**
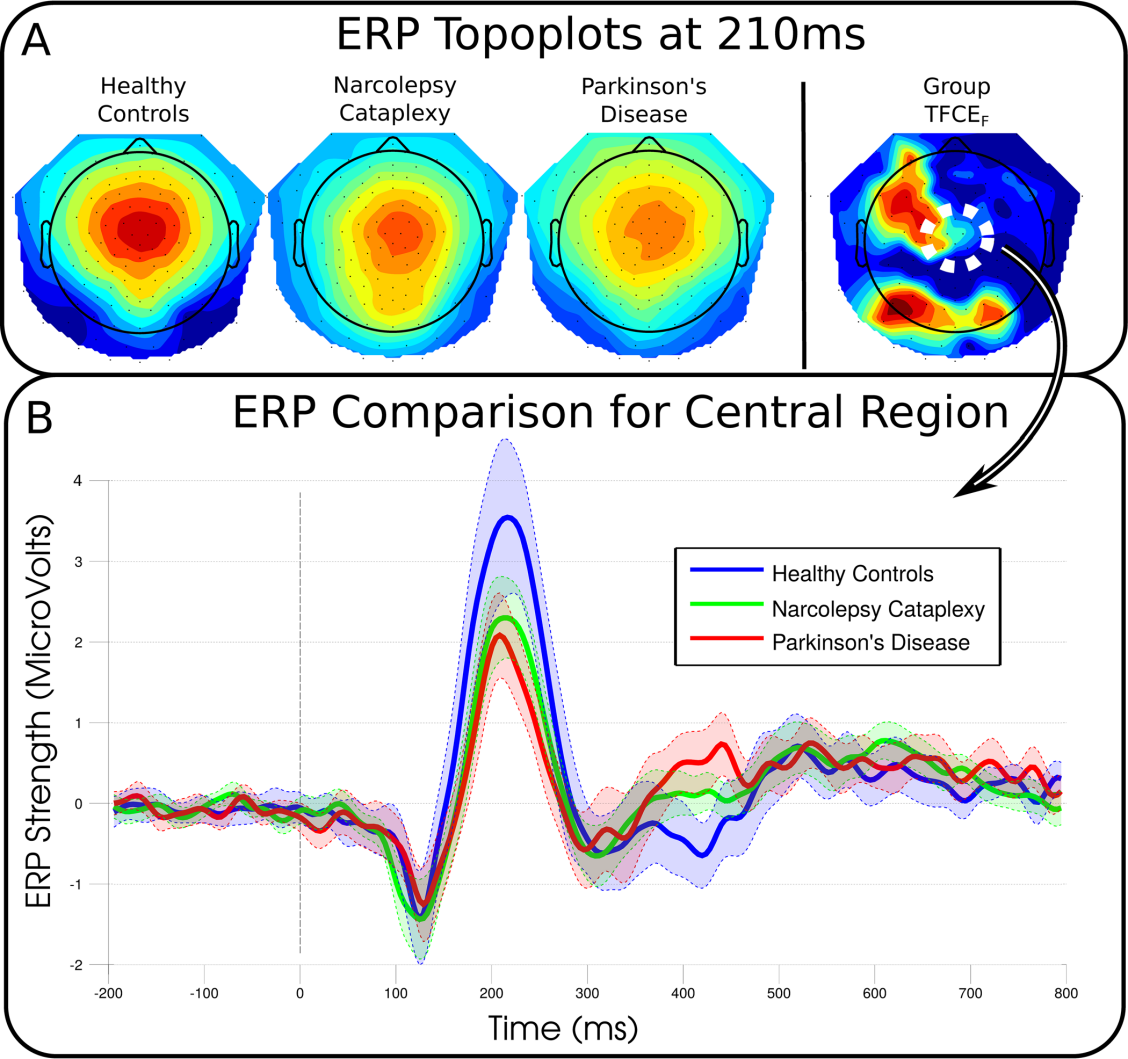
Cue-locked group topographies and event-related potentials over a central region of electrodes. No significant differences were found for either the group comparisons (12 participants in each group), or cue-type (valence or magnitude), conditions indicating participants‘ and patients‘ brain reactions were essentially the same for any cue. A, Topographies of the each group average and the resulting statistical comparison (blue to red as increasing significance). B, Event-related potentials recreated from the mean of 8 neighbouring channels in the central region of the scalp indicated by a dashed white circle in the group topoplot (E6, E7, E13, E30, E105, E106, E112, E129). The differences in groups around 210 ms were not found to be significant (F_2,33_ = 2.245, p = 0.94), but individual participants amplitudes' were predictive of the early feedback-locked ERPs.

Fig 2 shows the activity of the central region and the most prominent ERP elicited by the cue. The cue events clearly generated reliable ERPs with consistent topographies, but these did not statistically differ between groups or conditions. Group differences peaked at channel E19 (left fronto-central channel) at 768 ms (F_2_,_33_ = 12.940, p = 0.171). ERPs between high and low cues varied maximally at 304 ms around channel E105 (right central; F_2_,_33_ = 19.221, p = 0.114).

### EEG: Feedback Event

**Fig 3:**
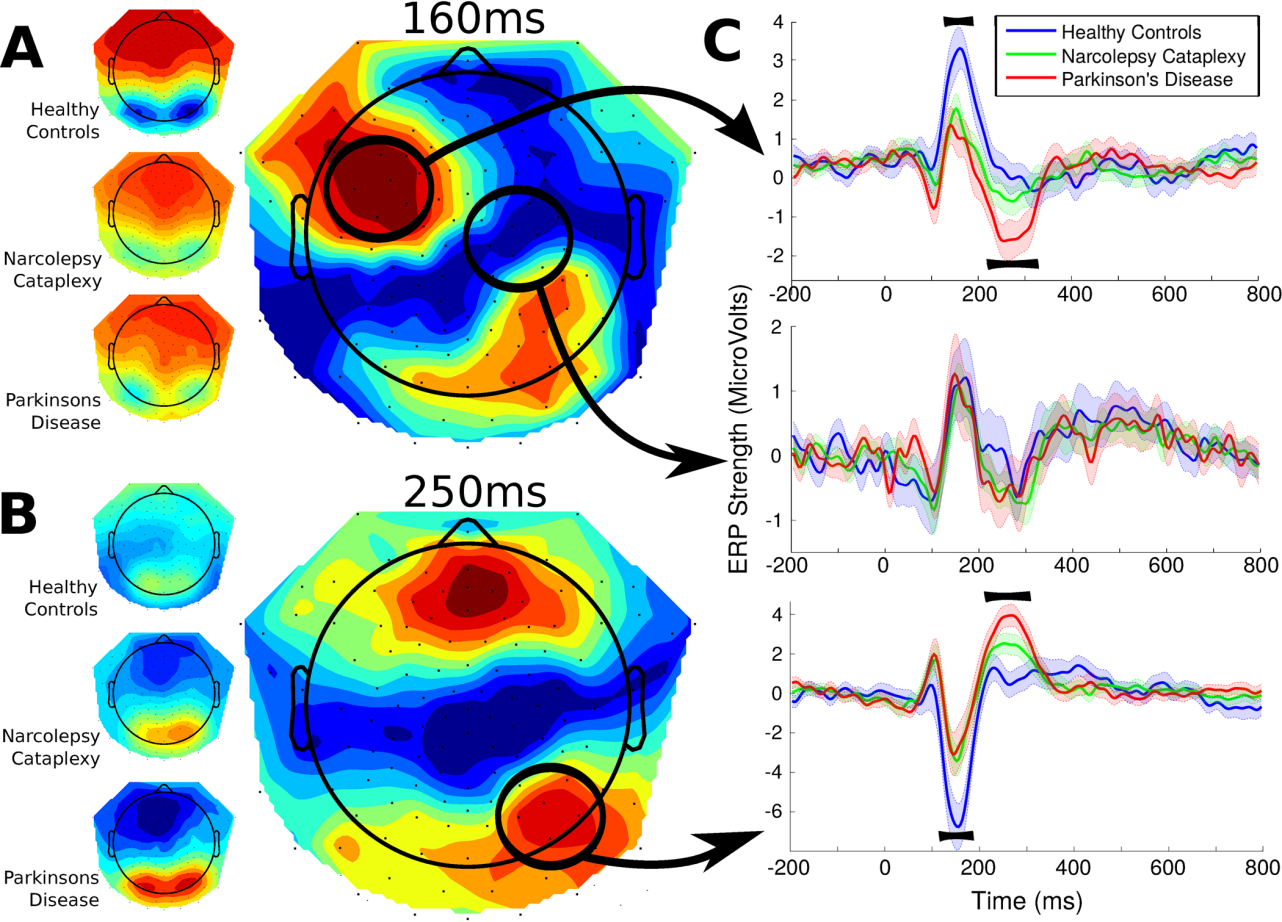
Feedback-locked group topographies and statistics at 160 and 250ms and Event-Related Potentials over right-posterior channels (the mean of 8 channels in the region). Reward feedback could indicate a successful or unsuccessful trial. Significant differences were found around two principle time points indicated by the topographies. A, The first difference showed similarly low amplitudes for the two patient groups compared to the healthy controls and may correspond to the N170 (12 participants in each group). B, The second, later difference showed an ERP for patient groups which was virtually absent in healthy controls. Additionally, significantly larger amplitudes for this component were found for Parkinson’s patients compared to participants with Narcolepsy-Cataplexy. These differences may correspond to feedback-related negativity (FRN) C, Three individual time courses of the entire event-related potential. The top and bottom panels both show the positive and negative deflections of the two topographies, while the middle panel indicates a time course where no significant differences were found for reference.

Significant main effects for group comprised of two main time windows; summarized in Fig 3. The first significant cluster of results was found around 160 ms over left fronto-central electrodes (peak: channel E34 at 168 ms; F_2_,_33_ = 11.390, p = 0.013). The later significant cluster of group effects ranged from 240 to 280ms and peaked over fronto-central regions around 260 ms (peak: channel E16 at 256 ms; F_2_,_33_ = 21.932, p < 0.001). Distinct from the earlier component, this later difference was a negative-going ERP on frontal channels with a corresponding positive-going ERP for posterior electrodes (peak: channel E91 at 268 ms; F_2_,_33_ = 16.622, p = 0.013). All other contrasts using more general conditions (i.e., all positive versus negative feedback trials, or high versus low magnitude stimulus), resulted in essentially identical group main effects, and none resulted in main effects of condition or significant interactions.

Post-hoc analysis to further examine the early (around 160 ms) and later (250 ms), group differences found using the TFCE_ANOVA_ was conducted by means of a repeated measures ANCOVA using the ERP amplitudes from both the gain and loss conditions as dependent measures, group as the between-subject factor, and the constructs of age and ESS as covariates. A right, posterior region of interest was selected which was significant for both early and later components and amplitudes were averaged over this area (channels E83, E84, E89, E90, E91, E95, E96 for the time window 132–180 ms and 244–280 ms for the early and later components respectively; see Fig 3). For the early component, only the effect of group was found to be significant (F_2,31_ = 6.540, p = 0.004); no other factor or interaction reached trend levels in this post-hoc comparison. Planned group contrasts confirmed that HC differed from both the NC (p = 0.004) and PD group (p = 0.003), but the patient groups did not differ from each other (p = 0.899), as apparent in Fig 3.

When examining the later component using post-hoc contrasts, we also included the amplitude values of the earlier component as a covariate to determine whether the effects related to one another. Once again, only the main effect of group was found to be significant (F_2,30_ = 11.909, p < 0.001). Importantly, the degree of individual variation in the later component was independent of the earlier component (F_1,30_ = 0.054, p = 0.818). Here, as before, HC significantly differed from both NC (p = 0.008) and PD patients (p < 0.001). Moreover, both the patient groups significantly differed from one another as well (p = 0.025), with PD patients showing the highest ERP amplitude and NC patients falling between PD and HC.

As an additional test, in accordance with previous findings of healthy controls [25], we used the mean amplitude values of the channels around the central peak from 200 to 230 ms of the cue as an individual marker of the emotional saliency (Fig 2), and included this additional factor as a predictor of the activity of both the early and later components in reward feedback. As in the reduced model described above, age* and ESS* were not significant predictors of early feedback amplitude, and group remained significant, albeit attenuated (F_2,30_ = 3.467, p = 0.044). Importantly, the mean amplitude of the cue-related potential was the strongest predictor of early feedback activity (F_1,30_ = 10.920, p = 0.002). The addition of the cue-related predictor had essentially no impact on the outcome for the later feedback component with only Group remaining significant (F_2,29_ = 8.840, p < 0.001). Since the association is found for the averaged ERPs and not the individual EEG time course, it is important to note that we cannot conclude whether the variability of the cue is causally related to the activity of the subsequent feedback, or whether it is a simple correlation reflecting a common source.

### The Effect of Medication in Narcolepsy-Cataplexy

Given that our NC population was on various medications, a 2x2 ANOVA using TFCE correction was performed on NC subgroups and feedback outcome (real gains versus real losses). Patients were divided into 3 equal sized groups of 4 patients in each group depending on whether they were un-medicated or primarily receiving treatment with Modafinil or Sodium Oxybate. No main effects or interaction with NC medication group was found for the channels and samples found to be significant earlier between HC and PD patients. The largest amplitude for these regions was 0.4 μV and topographies between each subgroup were consistent. However, despite the relatively low power in this analysis late significant group differences were found around 370 ms for bilateral temporal channels (peak: channel E119, at 372 ms; F_1,2_ = 64.37, p = 0.0328). During this time period, the non-medicated patients showed a positive central-posterior ERP component that was not present in either of the two medicated NC patient groups. In the larger context, non-medicated NC patients showed similar ERP amplitudes and topographies to HC over this time period, and after further analysis it was confirmed that only the medicated NC patients differed significantly from HC (T_11_ = 5.248, p = 0.045) at these later time points.

## Discussion

Here we identify differences during distinct stages of reward processing for NC and PD patients as compared to HC implicating distinct roles for dopamine and hypocretin during temporal processing of reward. While electrophysiological response during reward anticipation was similar in all groups, patients showed differences at two distinct stages of feedback processing, irrespective of valence and magnitude of feedback. These electrophysiological data enrich our understanding of the time course of HCRT dependent dysfunctions in the dopaminergic meso-cortico-limbic pathways, as had been shown for fMRI in NC [12]. In particular our findings suggest that both impairments to the HCRT and dopamine system initially induce a general pattern of early impairment and differentiate in function only in the later stages of reward processing.

### Reward Anticipation

There were no significant group or condition differences in EEG during the presentation of the cue. Yet, when presented with the same stimuli in our fMRI task, NC patients showed reduced VTA response (compared to HC) [12]. Although both EEG and fMRI generally show correlations as their signals both ultimately stem from neural activity, they are sensitive to different aspects [26,27]. When compared to other electrophysiological studies, the ERPs we obtained are strikingly similar to a recent study in HC [25], which compared rewarding cues to non-rewarding cue conditions and found lower amplitude responses around 220 ms for non-reward cues. Visual inspection of Fig 2 shows that in our data both patient groups did indeed show reduced amplitude around this time period, albeit not significant when corrected for multiple comparisons. Doñamayor and colleagues previously suggested that differences found in the response to the cue generally reflect the emotional saliency of the stimulus [25,28]. Moreover, they showed a linear relationship to cue magnitude and since our study did not use a completely non-rewarding cue, even low magnitude cues used here would have induced an emotional response, and the contrast between magnitudes is relatively weak. Moreover, Holroyd et al. [29], suggested that differential response to cue magnitude was a learned response, and as such may depend on a clear association between behavior and outcome. However, our task did not involve any learning, since we adjusted the difficulty of the task online to ensure an even number of successful and unsuccessful feedbacks, thus potentially leading to the non-significant group differences in response to the cue.

### Reward feedback

The early group difference in feedback ERPs peaked around 160 ms, which distinguished HC from both patient groups, likely reflects the electrophysiological activity associated with the VTA and/or NAc, which is disturbed in both patient groups [12]. Both the latency and topography of this ERP are reminiscent of the N170 component classically studied in the processing of faces [30]. Baker and Holroyd [31] found a ‘topographical N170’ which was sensitive to spatial cues in a maze-reward task, and whose amplitude was influenced by whether the stimulus was perceived to be informative, or not, of the final reward. In a similar fashion, the group differences for the N170-like component found here may reflect the patients’ sensitivity to whether feedback was perceived as informative. Support comes from a recent study in which the amplitude of the classic face N170 was modulated by perceived task relevance [32]. Thus, it seems both patient groups regard the feedback as less informative or relevant than HC. We further found that this component was significantly related to the individual variability of the amplitude of the cue-related ERP, described as a reflection of the emotional saliency of the reward cue in a previous study [25]. Thus, if the participant considers the cue information as less emotionally salient, then the feedback is also likely to be seen as less informative. The earlier feedback component was shown to be statistically unrelated to this later component and therefore the immediate underlying causes for the observed group differences are likely to be independent.

The ERP component around 250 ms showed the highest amplitude values for PD patients, followed by NC patients, and was essentially absent in HC. The topography of the component showed a large negative deflection over fronto-central electrodes, with posterior positivity. The topography and latency of the component are consistent with that of the feedback-related negativity ERP (FRN). Classic theories of the FRN suggest this ERP is only present for negative or aversive feedback [29]. However, recent theories suggest that classic experiments may have confounded feedback valence with expectedness, and that the FRN, and the anterior cingulate cortex from which it originates, more likely relates to the prediction of likely outcomes and signals when events depart from the previous prediction [33–36]. According to this predicted-response outcome (PRO) model account of the FRN, the generation of the error signal is a function of the *absolute* difference between reward predictions and the actual reward given over consecutive time steps [37]. In the case of HC, predictions of their outcome matched subsequent feedback. Thus, no error signal was generated, and no FRN signal is to be expected in their ERP [38]. Yet in our patient groups, if predictions are not formed, or are formed improperly, it would inevitably lead to a subsequent mismatch between prediction and outcome. No specific condition effect for the valence of the feedback would be expected in this account as the online tracking algorithm ensured an approximate 50% correct/error rate and so neither feedback type is inherently less expected.

Recent evidence from combined fMRI/EEG analysis supports several aspects of this account. Firstly, studies have found that the FRN is more likely to represent signals of absolute levels of surprise (positive or negative deviation from the expected), and is relatively independent of outcome valence [39]. Secondly, and most importantly here, dopaminergic midbrain regions send their reward prediction signals directly to the anterior cingulate cortex (ACC), the primary source of the FRN [14], and not via any other secondary structures [39]. Considering this model, dysfunction within regions of the mesolimbic dopaminergic circuit directly due to PD or indirectly in the case of NC, may result in altered computation of reward signals sent to the ACC. As tonic and phasic *changes* of dopamine input are more critical than absolute dopamine levels at eliciting activity in the ACC, both the dopamine deficit and the dopaminergic medication in PD-patients will generate incorrect predictions [40]. Accordingly, increasingly inaccurate predictions will tend to elicit larger prediction errors, resulting in increased ACC activity and thus larger FRN amplitudes; regardless of the valence of the prediction. Although the task used in this experiment did not involve any complex learning component (each cue type was associated with a fixed probability of winning), increased FRN reported here is compatible with findings of reward-based learning impairments found in PD [41–43]. NC patients also showed a clear FRN response to reward feedback, but with lower amplitude than the PD patients. This may be interpreted as an inconsistent FRN which produces a weaker averaged ERP response, or a more accurate predicted reward in NC producing consistent but weaker FRN amplitudes. Reversible inhibition of ACC activation has been recently shown to suppress cataplexy in hypocretin knock-out mice; pointing to the crucial role of ACC activation in mediating cataplexy via downstream motor pathways [44]. Although none of the NC participants showed clear signs of cataplexy during this reward-based experiment, our finding that NC patients showed an FRN response independent of valence is consistent with the clinical observations that cataplexy is triggered by both positive and negative emotions, as well as those related to unexpectedness or mere surprise [45]. Therefore, we propose that this impairment in predicted-outcome model of reward, leading to activation of the mPFC/ACC, the primary source of the FRN response, is a potential mechanism involved in the promotion of cataplexy in patients with NC.

### Limitations

It may be initially tempting to attribute group differences in ERPs to age and vigilance [46,47], as both these factors differ between groups. However, on a purely statistical level, substantial within-group variations on both these factors had no relationship with the EEG measures. That is, if groups were examined independently, the variation in age nor ESS scores was not a significant predictor of the either the early or late ERP amplitudes. Moreover, ERP amplitude differences were largest between PD patients and controls, with NC patients in between, despite the fact that NC patients had the lowest vigilance levels. Similarly with age, NC and PD patients either do not differ in their ERPs (early peak) despite having the largest age difference, or PD patients actually had higher amplitudes, followed by the NC patients groups in comparison to controls.

The unknown effect of medication in our patient populations limits the interpretation of the underlying causes. Although we found no effects of medication within the NC group, the sample sizes are small for such analyses to be conclusive. On the other hand, almost every PD patient we tested remained on their prescribed, dopaminergic medication during the time of the experiment. As such, it is impossible to determine whether the results found are attributable to the inherent pathology of PD, or induced by the increases in available extracellular dopamine attributed to these medications [2]. Indeed, one cannot even know with medication if the effect was sufficient to compensate for the loss of available dopamine in reward structures, or even act as a source of impairment under the ‘overdose hypothesis’ which holds that sufficient dopaminergic medication to control motor symptoms could actually lead to impairment for other functions [48]. Yet without extensive study of each individual PD patients' progression in pathology, the same doubt about the level of available dopamine in the mesolimbic structures and its behavioural consequences can be made of even drug-naive patients. Thus, although we cannot delineate the exact contribution of altered dopaminergic tone to the FRN, our results strongly suggest that any impairment in dopaminergic modulation, both under and over activation, would likely result in inadequate expectation signaling and thus the FRN response found here. Future studies may address this issue using drug-naive patients, although previous studies have indicated little effect of medication on reward tasks [42,49].

### Conclusion

Here we presented direct evidence for electrophysiological differences during reward processing for NC and PD patients as compared to HC. For the first time to our knowledge both NC and PD patients as well as healthy controls were included in the same study design to better understand the respective contribution of the HCRT and dopamine system in basic reward processing. Although both sleepiness and age are inherently linked to the conditions of NC and PD, we show that these factors do not predict the observed differences in reward processing. Instead, we show that these differences in the patients are specific to the patient groups at hand and thus to HCRT and/or dopamine dysfunction. Our results suggest that both patient groups were unable to generate accurate predictions about the reward feedback they received. PD patients, with their impairment directly related to the dopaminergic system showed the largest error-prediction response, as indexed by the FRN. NC patients showed an increased FRN relative to controls, but less marked compared to PD patients, indicating HCRT is an indirect influence on dopaminergic structures and its deficiency leads to impairment on a smaller scale compared to impairments to dopamine itself.

## Supporting Information

S1 Fig The effects of independent component analysis for the removal of artifacts present in the raw electroencephalographic (EEG) signal. Red lines indicate the original filtered signal while the black lines indicate the same signal after specific component removal. Each part shows two nearby channels, one affects by the artifact and the other not. As can be seen, the none-affected channel shows minimal changes to the raw signal after removing artifactual components. A (top) shows specific tremor movement artifacts found in the majority of patients with Parkinson’s disease. B (middle), shows clear electrocardiographic (ECG) artifacts. C (bottom) indicates muscle activity artifacts similar to electromyographic (EMG) recordings.
(TIF)
