# Supplementary figures and images for: The Roles of Dopamine and Hypocretin in Reward: A Electroencephalographic Study
Source: PLoS One. 2015 Nov 23;10(11):e0142432. doi: 10.1371/journal.pone.0142432 (PMC4658140; doi:10.1371/journal.pone.0142432)

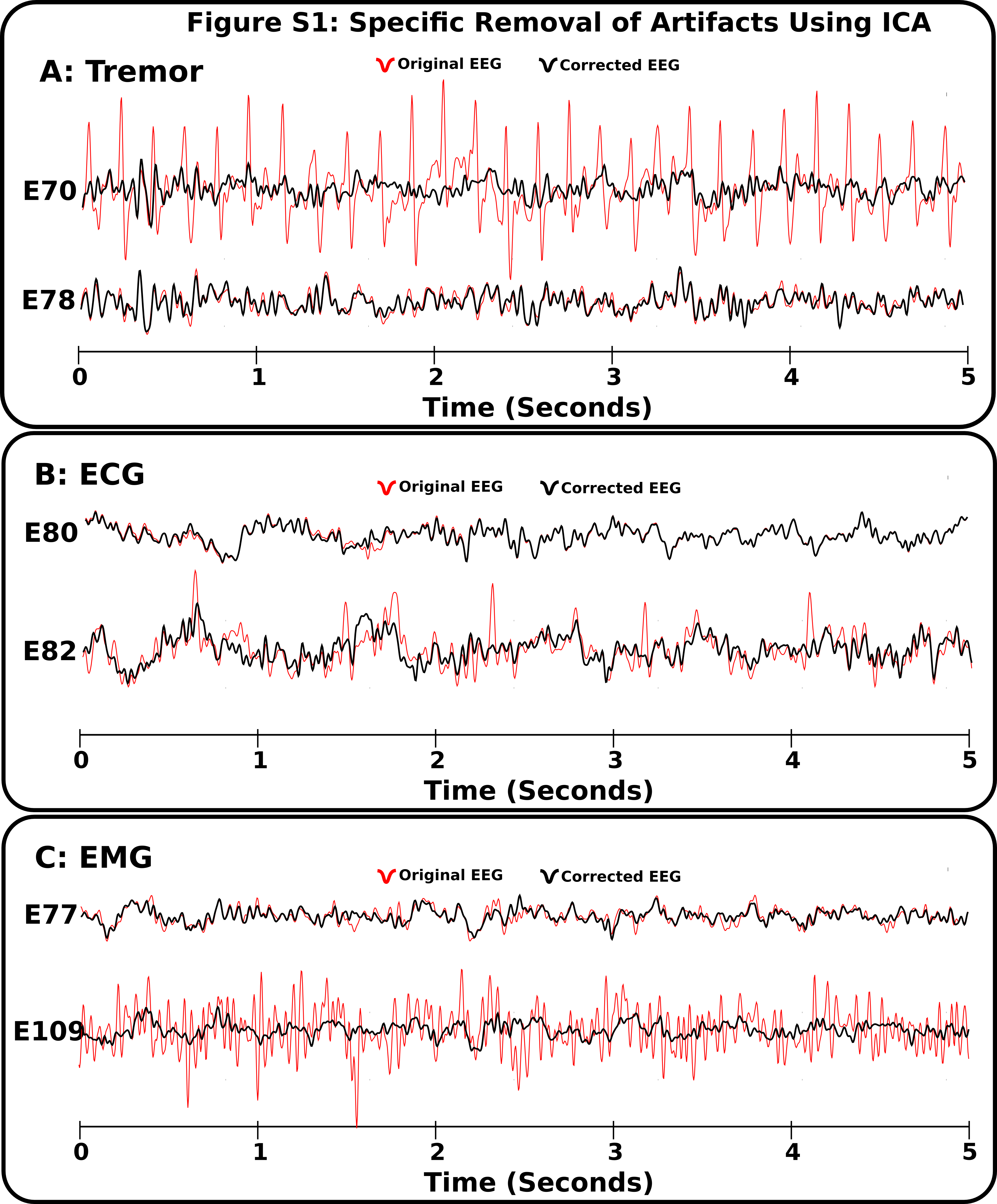

Supplement: S1 Fig — Red lines indicate the original filtered signal while the black lines indicate the same signal after specific component removal. Each part shows two nearby channels, one affects by the artifact and the other not. As can be seen, the none-affected channel shows minimal changes to the raw signal after removing artifactual components. A (top) shows specific tremor movement artifacts found in the majority of patients with Parkinson’s disease. B (middle), shows clear electrocardiographic (ECG) artifacts. C (bottom) indicates muscle activity artifacts similar to electromyographic (EMG) recordings. (TIF) [file pone.0142432.s001.tif]
